# Supplementary material for: Easily Synthesized Polyaniline@Cellulose Nanowhiskers Better Tune Network Structures in Ag-Based Adhesives: Examining the Improvements in Conductivity, Stability, and Flexibility
Source: Nanomaterials (Basel). 2019 Oct 30;9(11):1542. doi: 10.3390/nano9111542 (PMC6915529; doi:10.3390/nano9111542)
Supplement: Supplementary file 1 [file nanomaterials-09-01542-s001.pdf]

Supporting Information

Easily Synthesized Polyaniline@Cellulose Nanowhiskers Better Tune Network Structures in Ag-Based Adhesives: Examining the Improvements in Conductivity, Stability, and Flexibility

Ge Cao [1,2], Xiaolan Gao [2], Linlin Wang [2], Huahua Cui [2], Junyi Lu [2], Yuan Meng [2], Wei Xue [1,2], Chun Cheng [2], Yanhong Tian [1,*], and Yanqing Tian [2,*]

[1] School of Materials Science and Engineering, Harbin Institute of Technology, Nangang District, Harbin 150001, China; 11749286@mail.sustc.edu.cn (G.C.); 11849284@mail.sustech.edu.cn (W.X.)

[2] Department of Materials Science and Engineering, Southern University of Science and Technology, Xili, Nanshan District, Shenzhen 518055, China; gaoxl@mail.sustc.edu.cn (X.G.); 11749154@mail.sustech.edu.cn (L.W.); cuihh3@mail.sustech.edu.cn (H.C.); 11612916@mail.sustech.edu.cn (J.L.); 11611831@mail.sustech.edu.cn (Y.M.); chengc@sustc.edu.cn (C.C.)

* Correspondence: tianyh@hit.edu.cn (Y.T.); tianyq@sustech.edu.cn (Y.T.)

Table of Contents

Figure S1. A specialized stretching-system for bending the ECAs sample.................Page S2

Figure S2. The structure of the silver flakes used in this study……..........................Page S2

Figure S3. Dispersion test of PANI@CNs (1:4) in water and ethanol……................Page S3

Figure S4. The current-voltage curves of the ECAs with different silver contents.....Page S4

Figure S5. The SEM of high-aspect-ratio PANI@CNs nanowhiskers……................Page S5

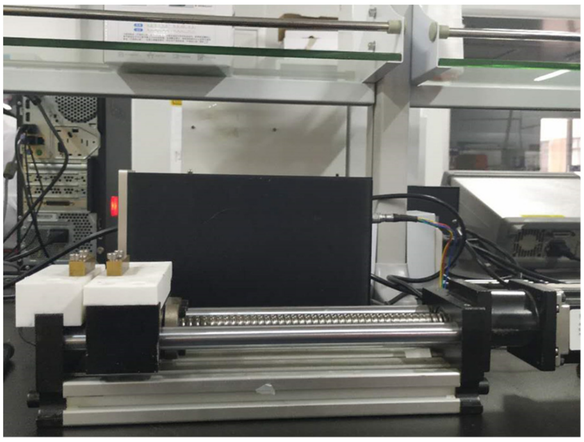

Figure S1. A specialized stretching-system for bending the ECAs sample.

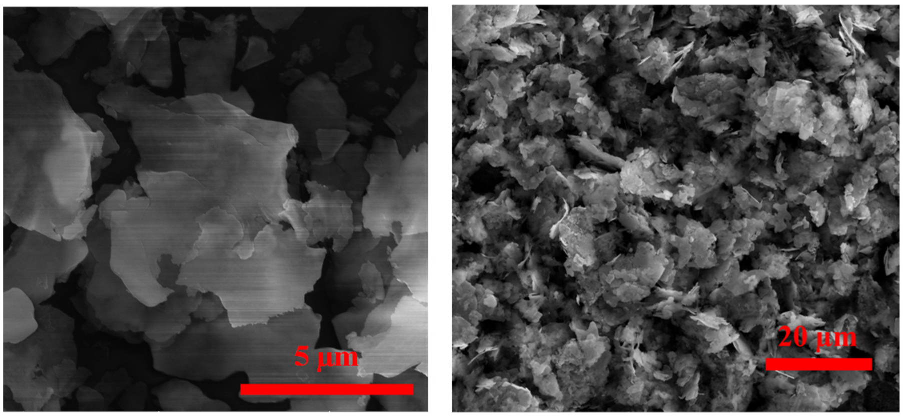

Figure S2. The structure of the silver flakes used in this study.

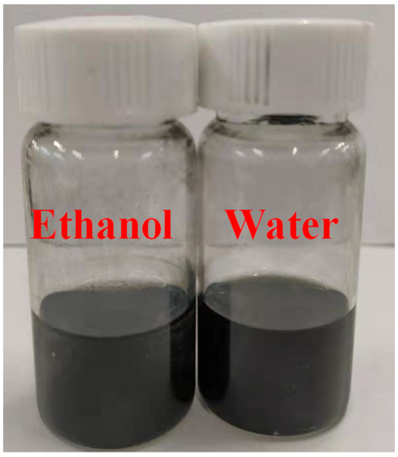

Figure S3. Dispersion test of PANI@CNs (1:4) in water and ethanol

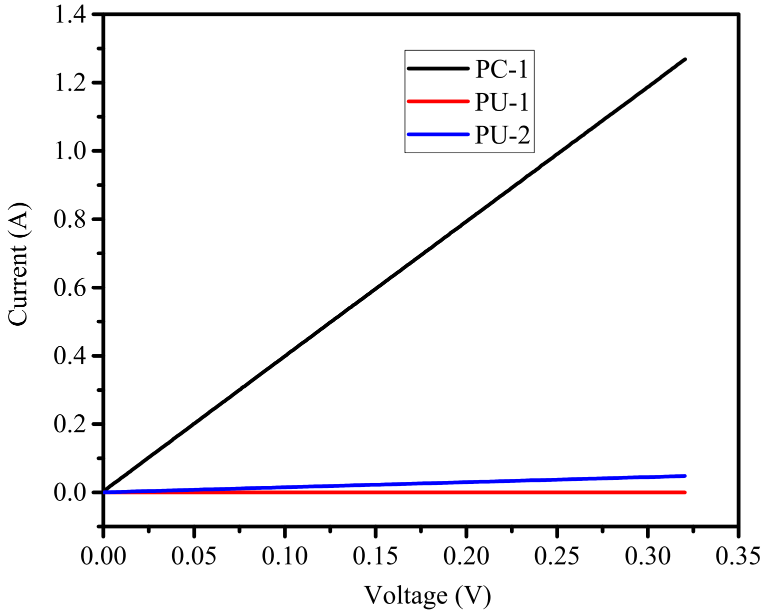

Figure S4. The current-voltage curves of the ECAs with different silver content.

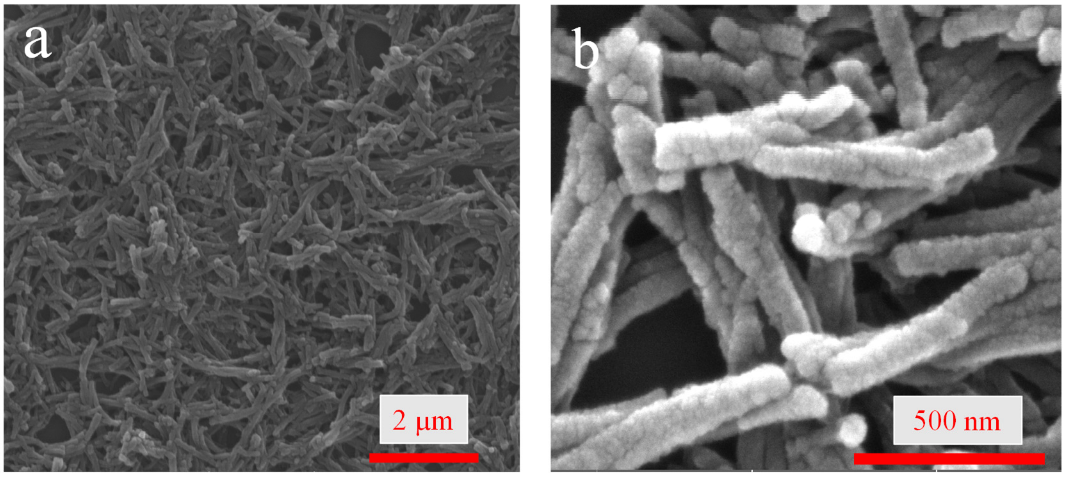

Figure S5. The SEM image of high-aspect-ratio PANI@CNs nanowhiskers

The percolation threshold of conductive fillers declined with an improvement of the aspect ratio (the ratio of length to diameter, L/D) of the fillers [1, 2]. For example, carbon black (CB) is one kind of spherical conductive carbon-materials for constructing conducting composites. Due to its lower aspect ratio (L/D≈1), a high fraction of CB (16%) was required to reach the percolation threshold. In contrast, CNTs with a much higher aspect ratio (L/D > 100) achieved much lower percolation thresholds (5%) [3]. Similarly, the PANI@CNs nanowhiskers (L/D > 10) had a high aspect ratio than common PANI particles (L/D≈1). Thus, PANI@CNs can exhibit better electrical properties than traditional PANI particles in the polymer matrix.

Reference

1. Munson-McGee, S.H. Estimation of the critical concentration in an anisotropic percolation network. *Phys. Rev. B* **1991**, *43*, 3331.
2. Dalmas, F.; Dendievel, R.; Chazeau, L.; Cavaillé, J.-Y.; Gauthier, C. Carbon nanotube-filled polymer composites. Numerical simulation of electrical conductivity in three-dimensional entangled fibrous networks. *Acta. Mater.* **2006**, *54*, 2923-2931.
3. Tang, H.; Chen, X.; Luo, Y. Electrical and dynamic mechanical behavior of carbon black filled polymer composites. *Eur. Polym. J.* **1996**, *32*, 963-966.
